# Supplementary material for: Compliance to food safety standards: Determining the barriers within the hotel industry
Source: PLOS Glob Public Health. 2025 Nov 21;5(11):e0002771. doi: 10.1371/journal.pgph.0002771 (PMC12637894; doi:10.1371/journal.pgph.0002771)
Supplement: S1 Text — (DOCX) [file pgph.0002771.s001.docx]

**DEMOGRAPHIC PROFILE**

**Please tick against the answer that best fits your response.**

1. Sex Male [ ] Female [ ]

2. What is your age? …………………….

3. What is your level of education? (Tick only one)

a. Primary school [ ]

b. JHS [ ]

c. Middle school [ ]

d. SHS [ ]

e. Vocational school [ ]

f. Polytechnic [ ]

g. University [ ]

h. No education.

4. What is your job title/position in the hotel?

a. Cook/chef [ ]

b. Assistant cook [ ]

c. Waiter/waitress [ ]

d. Storekeeper [ ]

e. Purchasing officer [ ]

f. Food and Beverage Manager [ ]

g. Other (Specify)……………………………………………….

5. How long have you been working in the foodservice Industry?

………………………………………….

6. How long have you worked this hotel?.....................................

7. Have you worked in the food service department of any hotel before joining this hotel?

Yes [ ]

No [ ]

8. If yes to question 6, where did you have your training? (Tick more than

one).

a. On the job [ ]

b. Apprenticeship [ ]

c. School [ ]

d. Workshop [ ]

e. Other specify……………

**FOOD SAFETY TRAINING**

1. Have been given food safety training since you were employed?

Yes [ ]

No [ ]

2a. In the year 2019 did have training on food safety

Yes [ ]

No [ ]

2b. In the year 2020 have had any food safety training?

Yes [ ]

No [ ]

If “No” to question 2a and 2b do not answer question 3, 4 and 5

1. If yes who organized it

a. FDA [ ]

b. Hotel management [ ]

c. Environmental health and sanitation

d. Others [ ]

1. Did you find it helpful?

Yes [ ]

No [ ]

1. Who paid for the training?
2. Hotel management [ ]
3. Self [ ]
4. Others **[ ]**
5. Are you aware of the FDA compliance code for Ghana?

Yes [ ]

No [ ]

1. Have you ever been trained on it?

Yes [ ]

No [ ]

| **Compliance with Food Safety FDA’S**  **Code for Food Hygiene Practices**  ***For each of the following items tick (√) “Yes” or “No” in the appropriate boxes as it applies to your hotel.*** | | | |
| --- | --- | --- | --- |
|  | | **Yes** | **No** |
| **a** | This hotel has adequate storage facilities for storing raw and cooked foods. |  |  |
| **b** | In this hotel thermometers are available for checking the |  |  |
| **c** | This hotel has spacious and well organized kitchen. |  |  |
| **d** | This hotel has changing rooms for employees. |  |  |
| **e** | This hotel has a wash room that is conveniently located and readily accessible for food service employee. |  |  |
| **f** | This hotel has a designated place for Employees to eat their meals. |  |  |
| **h** | This hotel has a designated sink for hand washing solely for food service workers with adequate handwashing materials. |  |  |
| **I** | In this hotel appropriate materials are used for surfaces and they are easy to clean and maintain |  |  |
| **j** | Walls, ceilings and floors of this hotel is made of light coloured and easy to clean materials. |  |  |
| **k** | The doors to washrooms, food preparation and service areas in this hotel is self-opening. |  |  |
| **l** | In this hotel food handlers use separate chopping boards for handling foods. |  |  |
| **m** | In this hotel, there is a sign/poster above every hand washing facility that reminds employees to wash their hands |  |  |
| **n** | In this hotel there are waste bins with fitting lids. |  |  |
| **o** | In this hotel the kitchen is well lit. |  |  |
| **p** | This hotel has a well-ventilated kitchen. |  |  |
| **q** | In this hotel, work surfaces are sound and in good condition (Rust free, smooth, not chipped). |  |  |
| **r** | This hotel has adequate supply of water. |  |  |

| **Barriers to Food Safety Compliances**  ***For each of the following items tick (√) “Yes” or “No” in the appropriate boxes.*** | | | |
| --- | --- | --- | --- |
|  | | **Yes** | **No** |
| 17 | Unconducive work environment in terms of structure (physical features). |  |  |
| 18 | Lack of motivation |  |  |
| 19 | Inadequate knowledge on what one is expected to do. |  |  |
| 20 | Lack of training and education on appropriate food safety practices. |  |  |
| 21 | Small working space |  |  |
| 22 | Poor monitoring and enforcement of regulations |  |  |
| 23 | Time pressure or busy work schedules. |  |  |
| 24 | Lack of necessary equipment and resources. |  |  |
| 25 | Inconvenient location of equipment such as sinks. |  |  |
| 26 | Work place policy/rules and regulations. |  |  |
| 27 | Food safety culture |  |  |
| 28 | Forgetfulness |  |  |

**BEHAVIOURAL OBSERVATION INSTRUMENT**

Date: _______________________________________

Observation Time: ___________ until _____________

Area Observed: _______________________________

**Instructions**:

Tick yes or no if the following are practiced:

|  | **Personal Hygiene** | **Yes** | **No** | **Remarks** |
| --- | --- | --- | --- | --- |
| 1 | Workers well groomed. |  |  |  |
| 2 | Proper clean attire worn. |  |  |  |
| 3 | Head gear worn /hair restraint worn. |  |  |  |
| 4 | Food handler washes hands with soap and warm water before and during food preparation and service |  |  |  |
| 5 | Food handler wears gloves during the preparation and serving of ready to eat foods or foods eaten raw. |  |  |  |
| 6 | Food handler washes hands in between handling raw and cooked food. |  |  |  |
| 7 | Food handlers do not wear jewelry or false nails, which might fall into food. |  |  |  |
|  | **Temperature Control** |  |  |  |
| 8 | Probe thermometer available. |  |  |  |
| 9 | Internal cooking temperature of food is checked. |  |  |  |
| 10 | Frozen food is thawed using acceptable methods (overnight in the refrigerator or in a container of cold water). |  |  |  |
| 11 | Cold food held at appropriate temperature <40^0^F/5^0^C. |  |  |  |
| 12 | Hot food held at appropriate temperature > 140^0^F/60^0^C. |  |  |  |
| 13 | Temperature of held food checked after every two hours. |  |  |  |
| 14 | Adequate refrigerators/freezers. |  |  |  |
| 15 | Cold room (4^0^C to 10^0^C) |  |  |  |
| 16 | Dry goods store (room temperature). |  |  |  |
|  | **Cross-contamination** |  |  |  |
| 17 | Ready-to-eat and raw foods are prepared separately. |  |  |  |
| 18 | Raw foods are stored below ready-to-eat foods in walk-in storage areas. |  |  |  |
| 19 | Work surfaces and utensils are sanitized after cutting raw food. |  |  |  |
| 20 | Raw foods are stored separately from the cooked foods. |  |  |  |
| 21 | Separate (colour coded if possible) chopping boards for cooked and uncooked foods. |  |  |  |
| 22 | Gloves when used are changed and disposed off after every use. |  |  |  |
| 23 | Equipment and serving dishes are washed and rinsed under running water |  |  |  |
| 24 | Serving plates and dishes are heated or sanitized before they are used for service |  |  |  |
|  | **Kitchen physical environment** |  |  |  |
| 25 | Separate sinks available for hand washing with hot (820C) running water and soap. |  |  |  |
| 26 | Hand drier or disposable towel available. |  |  |  |
| 27 | Separate work surfaces provided for different food items. |  |  |  |
| 28 | Work surfaces made of non-absorbent materials, inert to food, to detergent and disinfectants. |  |  |  |
| 29 | Work surfaces are clean and easy to clean. |  |  |  |
| 30 | The kitchen is well lit. |  |  |  |
| 31 | The kitchen is well ventilated. |  |  |  |
| 32 | Work surface is in sound condition (smooth, not chipped and with no rust). |  |  |  |
| 33 | Colour of work surfaces ensures easy cleaning. |  |  |  |
| 34 | Kitchen spacious and organized |  |  |  |
|  | **Facility Environmental Hygiene and Sanitation** |  |  |  |
| 35 | Doors, windows and other openings protected to eliminate pests |  |  |  |
| 36 | Hand washing stations have been equipped with sanitary towel or suitable drying service |  |  |  |
| 37 | Kitchen provided with self-closing doors |  |  |  |
| 38 | Food handlers operate in a clean environment (inside and outside) |  |  |  |
| 39 | Waste bins with Fitting lids available |  |  |  |
| 40 | Floors, Walls and Ceilings kept clean; free from dirt, stains and cobwebs |  |  |  |
| 41 | Adequate and appropriate drainage system provided |  |  |  |
| 42 | Provision of adequate toilet facilities and accessories for staff. |  |  |  |
| 43 | Toilet facilities kept clean and in a good state of repair |  |  |  |
| 44 | Provision of hand washing stations for use by kitchen staff |  |  |  |
| 45 | Hand washing stations have been equipped with sanitary towel or suitable drying service |  |  |  |

**INTERVIEW GUIDE FOR MANAGERS/SUPERVISORS OR CHEFS**

1. Is this your first place of work?
2. How long have you worked here?
3. What is your level of education?
4. In your opinion are foodborne illnesses a problem in Cape Coast and

Elmina? Explain

1. If so, what do you think are the causes?
2. What measures have you put in place to ensure food service staff comply with food safety standards in your hotel?
3. How many times in a year do calibrate the temperatures of your cold storage facilities?
4. Explain how you monitor the internal temperature of food when cooking? (use of a probe thermometer and if not, what happens).
5. Which foods do you consider to be highly hazardous? How do you handle them to keep them safe for consumption?
6. Which stages in the flow of food do you consider important in ensuring food safety? Give reasons
7. How do you ensure that all the staff in the food service sections is/are awareness of the importance of their role in ensuring food safety? (A in-service training seminars or workshops? Explain.
8. As food and beverage manager, what challenges do you face in ensuring food safety compliance in your hotel?

**INTERVIEW GUIDE FOR THE REGULATORY AGENCIES**

1. In your opinion are foodborne illnesses a problem in Central and Western Regions and Ghana? Explain

2. If so what do you think are the main causes?

3. Are foodborne diseases health issues or for compliance sake?

4. What measures have you put in place to ensure hotels comply with food safety standards?

5. What policies regulate food safety standards in hotels in Central and Western Regions?

6. What are the perceived barriers in enforcing these regulations?

7. Do you organize food safety training?

8. How often do you train on food safety? (Monthly, quarterly, annually)

9. Who are the targets of the training?

10. How do you disseminate information on food safety/food poisoning issues? (Television, radio,

newspaper, workshops)

11. How responsive are participants to the knowledge shared at the training workshops?

12. What are the challenges in disseminating food safety education?

13. What needs to be done to overcome these challenges?

14. If supervision is one of your major responsibilities in enforcing compliance to food safety

regulations, how many times is it carried out in a year?

15. What punishments do you give to hotels which do not comply with food safety standards?

| **Symmetric Measures** | | | | | |
| --- | --- | --- | --- | --- | --- |
|  | | | | | |
|  | | Value | Asymp. Std. Error^a^ | Approx. T^b^ | Approx. Sig. |
| Measure of Agreement | Kappa | .768 | .012 | 40.918 | .000 |
| N of Valid Cases | | 2835 |  |  |  |

| a. Not assuming the null hypothesis. |
| --- |
| b. Using the asymptotic standard error assuming the null hypothesis. |

| **Reliability Statistics** | | | |
| --- | --- | --- | --- |
| Cronbach's Alpha | Part 1 | Value | .807 |
|  |  | N of Items | 23^a^ |
|  | Part 2 | Value | .802 |
|  |  | N of Items | 22^b^ |
|  | Total N of Items | | 45 |
| Correlation Between Forms | | | .988 |
| Spearman-Brown Coefficient | Equal Length | | .994 |
|  | Unequal Length | | .994 |
| Guttman Split-Half Coefficient | | | .994 |

**Cronbach’s Alpha**

Part 1 Alpha = .807(23 items)

Part 2 Alpha = .802(22 items)

**Interpretation**

Both values are above 0.8, which indicates good internal consistency within each half of the test. This suggests the items within each part are reliably measuring the same construct.

**Correlation Between Forms** = 0.988

**Interpretation:**

This is the correlation between the two halves of the test. A value of 0.988 is very high, showing that both halves are measuring the same thing very similarly.

**Spearman-Brown Coefficient = 0.994**

**Interpretation:** This is another estimate of split-half reliability, less dependent on assumptions than Spearman-Brown. Again, 0.994 is excellent and confirms the instrument is very reliable.
